# Membranous Croup

**Published:** 1875-11

**Authors:** J. J. Maguigan


MEMBRANOUS CROUP: SHOWING THE EFFICIENCY OF MEDICINAL AGENTS BOTH IN THE CURE AND ARREST OF THE MALADY.—*By J. J. Maguigan, M.D.*— . . . I next tried a *hand-ball atomizer*, the kind used for perfuming handkerchiefs, and, filling it with lime-water, I played a beautiful spray into the fauces and larynx for at least five minutes. For the first time my efforts seemed to have met with a favorable response. In an easy manner this instrument accomplished the conjoint objects sought to be attained by the other two methods, viz: the application to the larynx of moisture and substance capable of acting chemically upon the membrane and dissolving it. The lime-water, which was the officinal solution, was used cold. The spray was employed half-hourly for the next twelve hours, and then hourly until convalescence was declared. The relief which it afforded, as indicated by the diminished dyspnœa, was always prompt and decided, particularly after the first few applications, when the membrane began to evince signs of rapid disintegration. The cough became less sonorous and more moist, and the expectoration free, the hawked-up matter being swallowed almost invariably. On the morning of the fifth day the larynx was entirely free. Respiration was entirely unimpeded, yet rough and croupal; the voice also remained husky, and a constant, short, laryngeal cough tortured him for the next twenty-four hours, for the relief of which a mixture of hydrate of chloral, tinct. opii, and ipecac was given. A simple catarrhal cough remained for two weeks or more.

So much with regard to the membrane, the expulsion of which may be considered the *sine qua non* to recovery; but scarcely less inimical to the life of the patient in this case was the superadded spasm. This element, though it played a subordinate role, would have determined the death of the patient but for the energetic manner in which its influence was combated by emetics and nauseants. By it all the frightful paroxysms of dyspnœa were superinduced. The most violent of these paroxysms, as well as a general exacerbation of the disease, invariably occurred at night on the second, third, fourth, and even the fifth night, the attack setting in at nightfall and subsiding at dawn of day. This nocturnal exacerbation and the prominence of the spasmodic element are two phenomena in the clinical history of this affection which have been fully portrayed by Jenner. . . . .

Lime-water is an active chemical solvent of pseudo-membrane. "We have frequently found," say Meigs and Pepper in their magnificent treatise on Diseases of Children, "when fragments of firm white exudation have been placed in lime-water at a temperature even lower than that of the buccal cavity, that the exterior began in a very short time (half an hour) to undergo disintegration, and that the whole fragment was reduced in a few hours to a granular putrilage." Oertel, describing the same process upon the toughest and most solid membranes he could find, says: "After the lapse of not more than fifteen or twenty minutes, particles of greater or less size gradually separated from the membranes, and after from thirty to forty-five minutes they are completely broken down, and, with a slight agitation of the test-tube, dissolved into a turbid, flocculent fluid."—*Philadelphia Medical Times.*

